# Antimicrobial Hypochlorous Wound Irrigation Solutions Demonstrate Lower Anti-biofilm Efficacy Against Bacterial Biofilm in a Complex *in-vitro* Human Plasma Biofilm Model (hpBIOM) Than Common Wound Antimicrobials

**DOI:** 10.3389/fmicb.2020.564513

**Published:** 2020-10-09

**Authors:** Julian-Dario Rembe, Lioba Huelsboemer, Isabell Plattfaut, Manuela Besser, Ewa K. Stuermer

**Affiliations:** ^1^Department of Vascular and Endovascular Surgery, University Hospital Düsseldorf, Heinrich-Heine-University of Düsseldorf, Düsseldorf, Germany; ^2^Chair for Translational Wound Research, Centre for Biomedical Education and Research, Witten/Herdecke University, Witten, Germany; ^3^Department of Vascular Medicine, University Heart and Vascular Center Hamburg, Translational Wound Research, University Medical Centre Hamburg-Eppendorf, Hamburg, Germany

**Keywords:** wound infection, biofilm, antimicrobial, antiseptic, hypochlorous acid, human plasma biofilm model, wound irrigation, chronic wound

## Abstract

Biofilms pose a relevant factor for wound healing impairment in chronic wounds. With 78% of all chronic wounds being affected by biofilms, research in this area is of high priority, especially since data for evidence-based selection of appropriate antimicrobials and antiseptics is scarce. Therefore, the objective of this study was to evaluate the anti-biofilm efficacy of commercially available hypochlorous wound irrigation solutions compared to established antimicrobials. Using an innovative complex *in-vitro* human plasma biofilm model (hpBIOM), quantitative reduction of *Pseudomonas aeruginosa*, *Staphylococcus aureus*, and Methicillin-resistant *S. aureus* (MRSA) biofilms by three hypochlorous irrigation solutions [two <0.08% and one 0.2% sodium hypochlorite (NaClO)] was compared to a 0.04% polyhexanide (PHMB) irrigation solution and 0.1% octenidine-dihydrochloride/phenoxyethanol (OCT/PE). Efficacy was compared to a non-challenged planktonic approach, as well as with increased substance volume over a prolonged exposure (up to 72 h). Qualitative visualization of biofilms was performed by scanning electron microscopy (SEM). Both reference agents (OCT/PE and PHMB) induced significant biofilm reductions within 72 h, whereby high volume OCT/PE even managed complete eradication of *P. aeruginosa* and MRSA biofilms after 72 h. The tested hypochlorous wound irrigation solutions achieved no relevant penetration and eradication of biofilms despite increased volume and exposure. Only 0.2% NaClO managed a low reduction under prolonged exposure. The results demonstrate that low-dosed hypochlorous wound irrigation solutions are significantly less effective than PHMB-based irrigation solution and OCT/PE, thus unsuitable for biofilm eradication on their own. The used complex hpBIOM thereby mimics the highly challenging clinical wound micro-environment, providing a more profound base for future clinical translation.

## Introduction

In wound management, controlling microbial bioburden is a key factor of prophylactic and therapeutic regimes. While wound contamination and colonization can mostly be handled with vigilance and mechanical cleansing, local infection with the potential threat of systemic spread requires antimicrobial intervention (IWII, 2016). Possible interventions range from preserved antimicrobials-containing wound irrigation solutions to antiseptics, debridement and systemic antibiotics in case of systemic spread. Biofilm formation thereby represents a specifically difficult to diagnose and manage complication in wound therapy. According to recent evaluations, over 78% of all chronic wounds are challenged by biofilm formation (Malone et al., 2017). Biofilms are structured communities of microorganisms attached to a surface (e.g., wound bed) encased within an extracellular matrix (ECM) referred to as the extracellular polymeric substance (EPS; Percival et al., 2015). Microorganisms embedded in the EPS demonstrate a significant tolerance and resilience to antimicrobial substances, biocides, and the host immunity due to a variety of factors such as polymicrobial heterogeneity, genetic diversity, and resistance transfer, dormant metabolism and the EPS itself functioning as a high-protein mechanical and diffusion barrier for antimicrobials (Hall-Stoodley and Stoodley, 2009; Hoiby et al., 2010; Percival et al., 2015; Salisbury et al., 2018). As a result, wound antimicrobials and antiseptics need to be thoroughly investigated regarding their anti-biofilm efficacy (Schultz et al., 2017).

Several *in-vitro* models have been applied for this purpose, growing wound biofilms on plastic or metal surfaces, in many cases without additional organic and/or human material (Brackman and Coenye, 2016; Schultz et al., 2017; Shukla et al., 2020). Such approaches are feasible for evaluating biocides and disinfectants for (surface) decontamination, but in case of wound antimicrobials, the more complex composition of the wound microenvironment (cytokines, proteases, fibrin, cells, etc.) with high-protein, organic challenge as well as the human immune system need to be considered. Therefore, our research group developed an innovative human plasma biofilm model (hpBIOM) for quantifiable testing of anti-biofilm activity of antimicrobial and antiseptic agents in a challenging wound biofilm environment, aiming to resemble the *in-vivo* situation (Besser et al., 2019, 2020).

Due to the recent renaissance of hypochlorous acid based antimicrobial wound irrigation solutions (Severing et al., 2019) with contrasting evidence regarding anti-biofilm efficacy, this study focused on the evaluation of these agents compared to established antimicrobial and antiseptic agents. Prolonged exposure times, increased substance volumes, and varying agents (with differing substance concentrations) were investigated using the hpBIOM and compared to a planktonic test setup to provide a comprehensive analysis.

## Materials and Methods

### Microbial Strains

*Staphylococcus aureus* (DSM-799) and *Pseudomonas aeruginosa* (DSM-939; both German Collection of Microorganisms and Cell Cultures (DSMZ), Braunschweig, Germany) as well as a clinical MRSA strain (provided by the Institute for Medical Laboratory Diagnostics, Helios University Hospital Wuppertal, Germany) were selected. All strains were previously tested for biofilm formation on plastic surfaces (data not shown). All strains were cultivated on casein/soy peptone agar plates (CSA) according to standard protocols and the second subculture was used for experiments.

### Antimicrobials and Antiseptics

Five antimicrobial solutions in total (Table 1) were evaluated. Three chlorine-based and -releasing agents, used as antimicrobial wound irrigation solutions: ActiMaris® forte [0.2% sodium hypochlorite (NaClO)/3% sal maris; AMF], Lavanox® (<0.08% NaClO; LVX), and Kerrasol® (<0.08% NaClO; KSL).

**Table 1:** Overview of the tested antiseptics and antimicrobial wound irrigation solutions, product specifications, manufacturer, and composition as specified by the manufacturer.

Test solution		Product	Manufacturer	Composition	Category
OCT/PE		Octenisept®	Schülke & Mayr GmbH	0.1 g octenidin-dihydrochlorid, 2.0 g 2-phenoxyethanol (per 100 ml)	Antiseptic
PHMB		Lavasorb®	Fresenius Kabi AG	0.4 g polyhexanide, 0.02 g macrogolum 4,000 (per 1,000 ml)	Antimicrobial irrigation solution
NaClO	AMF	ActiMaris®forte	ActiMaris AG	0.2% NaClO, 3% sea-salt, H_2_O	Antimicrobial irrigation solution
	LVX	Lavanox®	Serag Wiessner GmbH & Co KG	H_2_O, <0.08% NaClO	Antimicrobial irrigation solution
	KSL	KerraSol™	Crawford Healthcare GmbH	H_2_O, <0.08% NaClO	Antimicrobial irrigation solution

As reference substances, a polyhexanide (PHMB)-based antimicrobial irrigation solution (Lavasorb®; 0.04% PHMB) and the antiseptic Octenidine-dihydrochloride/phenoxyethanol (Octenisept®; 0.1% OCT/PE) were used. All products were handled in a sterile manner and according to manufacturer instructions.

### Neutralizing Agent and Dissolving Solution

To stop antimicrobial activity after specific designated exposure times, a neutralizing solution was used comprising of 40 g Tween 80, 30 g saponin, 4 g lecithin, 3 g sodium thiosulfate (all Carl Roth GmbH & Co. KG, Karslruhe, Germany) and 10 g sodium dodecyl sulfate (SDS; Caesar & Loretz GmbH, Hilden, Germany), and 500 ml double distilled H_2_O (ddH_2_O). The neutralizing solution composition was based on specifications from the standards (DIN EN 13727; DIN, 2013). Concentration, volume, and efficacy of the neutralizing solution have been preliminarily validated internally and demonstrated a sufficient neutralizing efficacy for all tested antiseptics and antimicrobials, non-toxicity toward microbial strains used in a planktonic, as well as a biofilm setup and showed no interference with the integrity of the biofilm model.

For dissolving the model after successful conduction of experiments to recover and quantify surviving microorganisms, a 10% (w/v) bromelain solution was used, as bromelain exhibits a fibrinolytic activity and has been previously used for debridement and biofilm dispersal purposes (Maurer, 2001; Pavan et al., 2012; Besser et al., 2019, 2020). Therefore, 10 tablets of Bromelain-POS® (500 F.I.P. units per tablet; URSAPHARM Arzneimittel GmbH, Saarbrücken, Germany) were dissolved in 100 ml phosphate buffered saline (PBS) and the solution stored at 4°C until further use. Bromelain was later added to the model in a 1:1 (v/v) ratio to the model volume (1.5 ml per model). Before usage, bromelain was warmed to 37°C for improved enzyme performance. The bromelain solution has been preliminarily tested and validated and showed no antimicrobial effect of its own.

### Human Plasma Biofilm Model Preparation

The development and use of the hpBIOM has been described in detail elsewhere (Besser et al., 2019, 2020) and was adapted to fit the specific agents and purpose pursued here. In brief, human plasma (citrate buffered) and buffy coat from anonymous donors were obtained from the DRK-Blutspendedienst West (Hagen, Germany). To remove as many residual erythrocytes as possible, the buffy coat was centrifuged at 3,000 rpm at room temperature (RT) for 30 min. Subsequently, plasma and buffy coat were merged, gently mixed in a sterile glass bottle, and continuously agitated at 22°C. A microbial test suspension was prepared by colony picking and adjusted to a 0.5 McFarland standard (~1.5 × 10^8^ cfu/ml) using a spectrophotometer (EON™; BioTek Germany, Bad Friedrichshall, Germany). Finally, a “master mix” was prepared by adding the appropriate amount of microbial test solution to the plasma and buffy coat mixture, resulting in a final bacterial concentration of ~1.5 × 10^6^ cfu per individual hpBIOM. Next, 18.26 μl calcium chloride (CaCl_2_; 500 mM) per ml plasma was added to the master mix, to induce fibrin polymerization, gently mixed, and immediately transferred into 12-well plates (1.5 ml per model/well containing plasma, buffy coat, and pathogenic bacteria). The plates were incubated for 12 h on a rotation shaker at 50 rpm and 37.0°C for the plasma solution to polymerize with pathogens rearranging and forming an ECM, finally yielding a stable biofilm disc/clot with integrated bacteria (~concentration of 1.5 × 10^7^ cfu/ml), EPS as well as human plasma and immune cells.

### Antimicrobial Treatment of the hpBIOM and Quantification of Bacterial Survival

After hpBIOM preparation, each clot was treated with 300 μl of antiseptic/antimicrobial test substance for 0.5, 2, 6, and 24 h. To additionally investigate the effect of prolonged exposure (in terms of remanence effect without reapplication) and/or increased substance volume (1 ml), experiments were additionally extended to 48 and 72 h with 300 μl or 1 ml of active substance against the clinically isolated MRSA strain and *P. aeruginosa*. After the specified treatment periods, antimicrobial activity was neutralized by adding 1.2 ml of the specified neutralizing solution to each well, detaching the model from the well walls with a pipette tip (in order to fully distribute the neutralizing solution around the model) and placing the plates on a rotation shaker at RT for 5 min ± 10 s. Subsequently, each hpBIOM was carefully transferred into a 15 ml falcon tube with 1 ml bromelain solution for dissolving the model. An additional 0.5 ml of bromelain solution was used to wash out the well to detach remaining microorganisms and added to the falcon tube.

In case of the 1 ml test setup, models were already detached and transferred into 15 ml falcon tubes for neutralization, due to the higher necessary amount of neutralizing solution (4 ml to keep a 1:8 ratio) and bromelain subsequently added to the tube after neutralization. After 2–3 h, the hpBIOM was completely dissolved. For quantification, the resulting solution was thoroughly vortexed, serially tenfold diluted, 50 μl of each dilution spread on agar plates and incubated overnight at 37°C under aerobic conditions. Remaining microbial counts after treatment were quantified by determining colony counts (in cfu/ml) using a Colony Counter Pen (eCount™, VWR Leicestershire, United Kingdom) and reduction rates calculated compared to an untreated control, as well as initial bacterial counts.

### Quantitative Suspension Method

To compare the anti-biofilm efficacy of the tested antiseptics and antimicrobials to the efficacy on planktonic bacteria, all tested substances were additionally evaluated using a quantitative suspension method (QSM). The QSM is based on DIN EN 13727 (DIN, 2013) and has been modified for direct comparison to the hpBIOM. Thereby, a bacterial test suspension was prepared by colony picking, adjusted to 1.5 × 10^7^ cfu/ml initial concentration (as for hpBIOM) and 1.5 ml of the prepared bacterial test suspension was treated with 300 μl of the respective antiseptic or antimicrobial for 0.5, 2, 6, or 24 h. To neutralize antimicrobial activity, 1.2 ml neutralizing solution was added and the samples subsequently incubated for 5 min ± 10 s on a rotation shaker at RT. Reduction rates (in cfu/ml) were quantified in the same manner as described for hpBIOM.

### Visualization of Biofilm Using Scanning Electron Microscopy

To visualize the morphology and structure of the bacterial biofilm with and without antiseptic/antimicrobial treatment, scanning electron microscopy (SEM) was performed for selected experimental setups (see Table 2). After neutralization of the antiseptic/antimicrobial agents, the models were fixed with a glutaraldehyde/PVP-solution containing 2.5% glutaraldehyde, 2% polyvinylpyrrolidone (PVP), and 0.5% sodium nitrite (NaNO_2_) in 0.1 M cacodylate buffer for 1 h at 4°C. Samples were washed in 0.1 M cacodylate buffer and stored at 4°C until preparation of freeze fracture fragments with liquid nitrogen. For glycocalyx staining, the samples were subsequently incubated in a solution containing 2% arginine-hydrochloride (HCL), glycine, sucrose, and sodium glutamate for 18 h at RT. The samples were rinsed with ddH_2_O and 0.1 M cacodylate buffer followed by immersion in a mixture of 2% tannic acid and guanidine-HCL for 5.5 h at RT. After another rinsing step with ddH_2_O and 0.1 M cacodylate buffer samples were incubated over night at 4°C. For staining, the samples were placed in a 1% osmium tetroxide (OsO_4_) solution for 30 min at RT. After three rinsing steps with 0.1 M cacodylate buffer, the samples were again stored over night at 4°C. Finally, samples were dehydrated using isopropyl alcohol and acetone and dried in liquid CO_2_ using a critical point dryer (BAL-TEC AG, Balzers, Liechtenstein). *Via* the sputter coater (BAL-TEC AG, Balzers, Liechtenstein), samples were sputtered with gold palladium and finally examined with a Zeiss Sigma SEM (Zeiss, Oberkochen, Germany) using 2 kV acceleration voltage and an inlens detector.

**Table 2:** Specification and overview of the experimental setups used for visualization of biofilm formation in the human plasma biofilm model (hpBIOM) using scanning electron microscopy (SEM).

Image	Setup/substance	Pathogen	Maturation time^*^	Treatment period
4 (a)	CTRL	*P. aeruginosa*	12 h	-
4 (b)	CTRL	*P. aeruginosa*	18 h	-
4 (c)	CTRL	*P. aeruginosa*	36 h	-
5 (a)	CTRL	*P. aeruginosa*	12 h	0 h
5 (b)	OCT/PE	*P. aeruginosa*	12 h	6 h
5 (c)	OCT/PE	*P. aeruginosa*	12 h	24 h
5 (d)	NaOCl (<0.08%)	*P. aeruginosa*	12 h	24 h

Figures 4A–C demonstrate a representative biofilm development over time. Figures 5A–D show the biofilm surface structure under treatment with antiseptics (OCT/PE) or antimicrobial wound irrigation solution (<0.08% NaOCl) over time compared to the initial structure.

* Before application of test substance, if any (not in case of CTRL).

### Statistical Analysis

All experiments were performed in triplicates with three different anonymous donors. Microbial reduction rates (in Δlog_10_ cfu/ml) were calculated for all tested antiseptic/antimicrobial solutions. High antimicrobial efficacy is defined as a reduction in initial microbial counts by at least 3 log_10_ reduction steps, as defined for antiseptics tested under organic challenge by national and international standards (DIN, 2013). Data are expressed as means ± standard error of the mean (*sem*) and were analyzed using the statistics program GraphPad PRISM (Version 8.2.1; GraphPad Software Inc., La Jolla, United States). Statistical analysis was performed using two-way ANOVA, followed by Holm-Sidak *posthoc* test for evaluation of multiple comparisons. A value of *p* ≤ 0.05 was considered statistically significant (^*^p ≤ 0.05; ^**^p ≤ 0.01; ^***^p ≤ 0.001; and ^****^p ≤ 0.0001).

## Results

### Antimicrobial Efficacy on Planktonic Bacteria (QSM) Within 24 H

**Figure 1:**
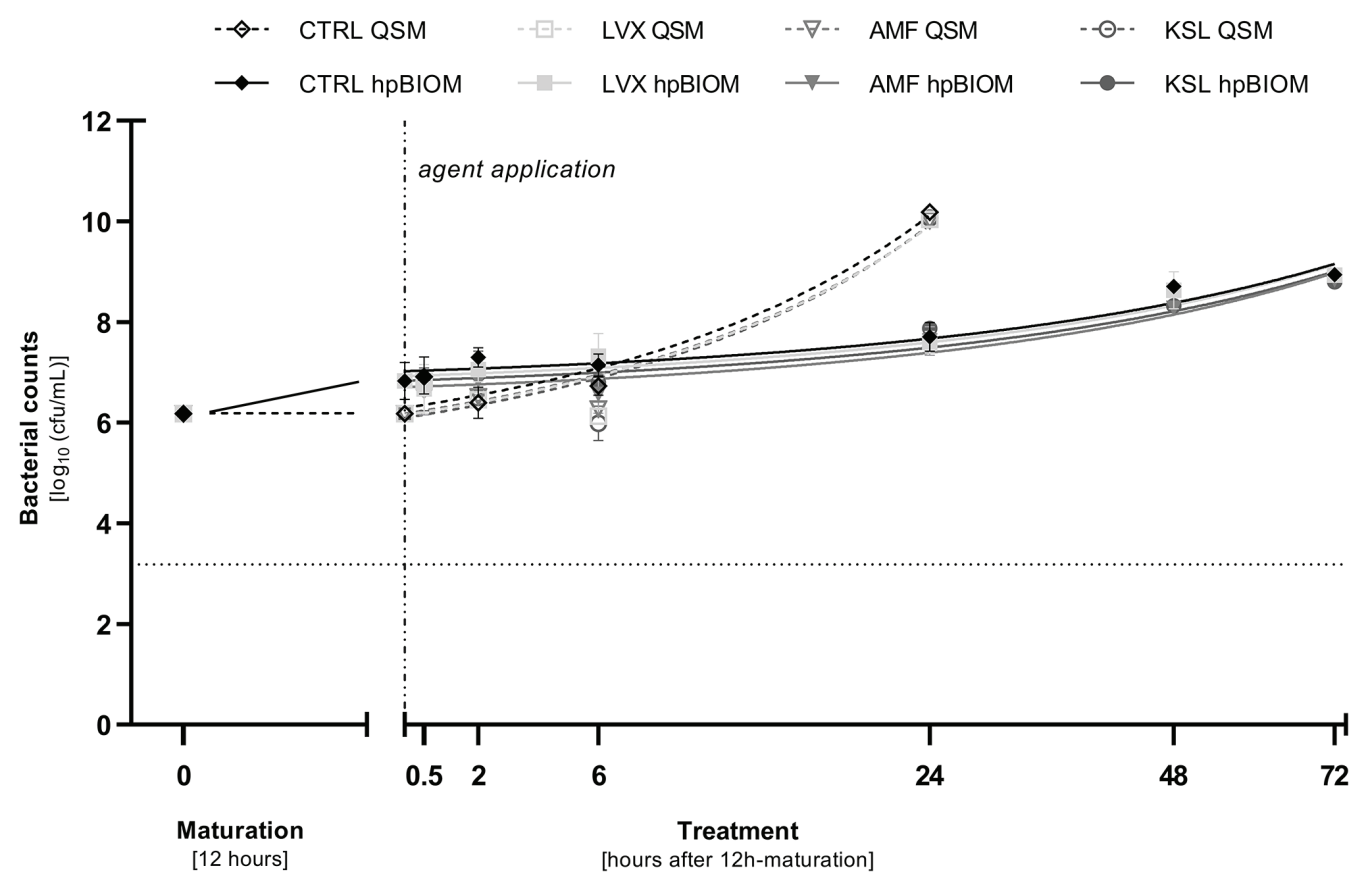
Reduction rates of 0.3 ml of tested antimicrobial irrigation solutions Lavanox® (LVX; <0.08% NaClO), Kerrasol® (KSL; <0.08% NaClO), and Actimaris®forte (AMF; 0.2% NaClO, 3% sal maris) compared to an untreated control (CTRL) in planktonic (QSM) and biofilm (hpBIOM) form (here against *Pseudomonas aeruginosa*). Bacterial counts (in log_10_ cfu/ml) are outlined over the course of 72 h of treatment after an initial biofilm maturation period of 12 h. Dotted horizontal line indicates the cut-off for a high antimicrobial efficacy (≥3 log_10_ reduction steps). Dashed vertical line indicates onset of treatment with the different antimicrobial agents after 12 h of biofilm maturation. Values are expressed as means ± standard error of the mean (*sem*) and time-kill-curves of bacteria in planktonic state (QSM) are only depicted for 24 h. All experiments were performed in triplicates.

On bacteria in a planktonic state, the three tested antimicrobial hypochlorous wound irrigation solutions containing NaClO/hypochlorous acid (HClO) demonstrated no bacterial reduction against any tested pathogen within 24 h (Figure 1).

**Figure 2:**
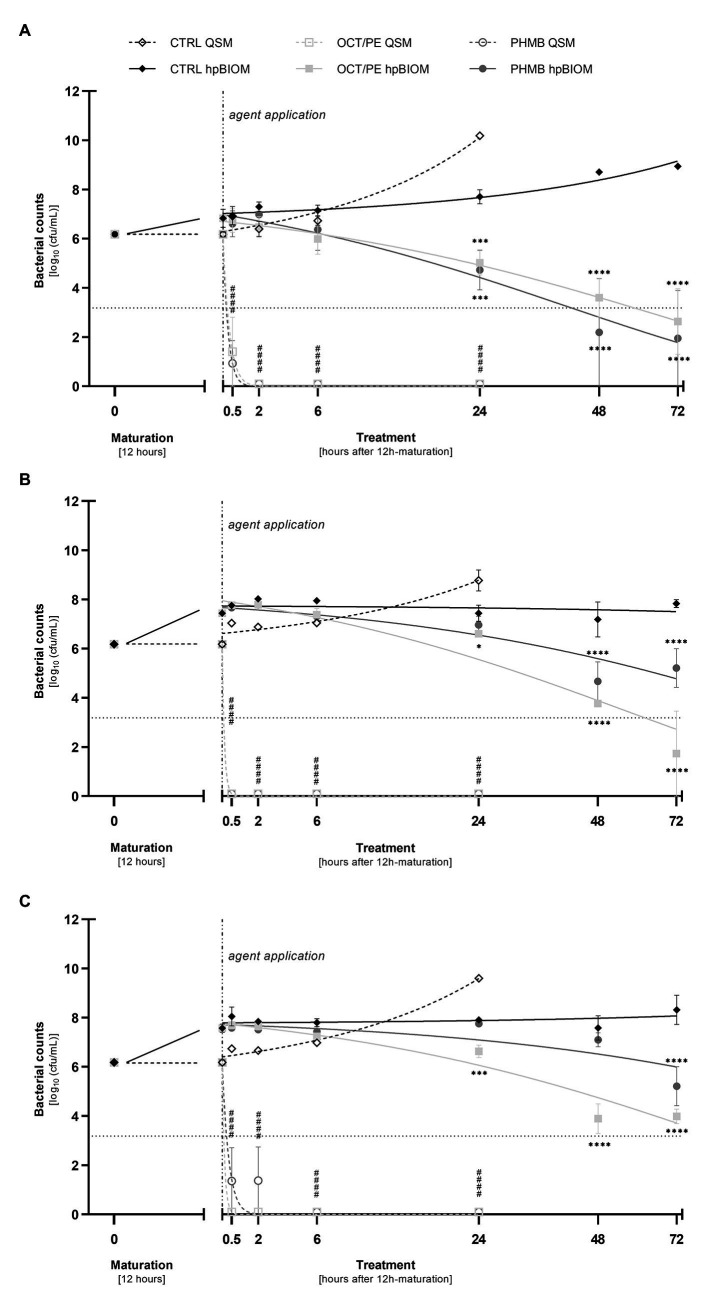
Reduction of *P. aeruginosa* **(A)**, MRSA **(B)**, and *S. aureus* **(C)** in planktonic (QSM) vs. biofilm (hpBIOM) form by 0.3 ml of tested antiseptics octenidine-dihydrochloride/phenoxyethanol (OCT/PE) and polyhexanide (PHMB) compared to an untreated control (CTRL). Remaining bacterial counts (in log_10_ cfu/ml) are outlined over the course of 72 h of treatment after an initial biofilm maturation period of 12 h. Dotted horizontal line indicates the cut-off for a high antimicrobial efficacy (≥3 log_10_ reduction steps). Dashed vertical line indicates onset of treatment with the different antimicrobial agents after 12 h of biofilm maturation. Values are expressed as means ± *sem*. Significant reductions compared to the untreated control are expressed as ^*^p ≤ 0.05, ^**^p ≤ 0.01, ^***^p ≤ 0.001, or ^****^p ≤ 0.0001 (# in case of QSM). All experiments were performed in triplicates [Time-kill-curves of bacteria in planktonic state (QSM) are only depicted for 24 h].

Both reference substances, OCT/PE and PHMB-based irrigation solution, achieved a highly significant reduction of *P. aeruginosa* (4.77 ± 1.41 and 5.25 ± 0.93 log_10_ steps, respectively; *p* < 0.0001), *S. aureus* (6.18 ± 0.00 and 4.82 ± 1.36 log_10_ steps; *p* < 0.0001), and MRSA (both 6.18 ± 0.00 log_10_ steps; *p* < 0.0001) within 30 min of exposure (Figures 2A–C). After 2 h of exposure, OCT/PE and PHMB both fully eradicated all three tested pathogens, except PHMB against *S. aureus*, needing 6 h for complete eradication (Figure 2C).

All reduction rates of the QSM are summarized in Supplementary Table 1.

### Anti-biofilm Efficacy (hpBIOM) Within 24 H

As in the QSM, the tested hypochlorous wound irrigation solutions showed no antimicrobial/anti-biofilm activity in the complex biofilm model (hpBIOM). No bacterial reduction could be observed against any tested pathogen within 24 h (Figure 1).

The tested reference substances showed a reduced effect within 24 h: against a methicillin-resistant (MRSA) as well as a methicillin-susceptible (MSSA) *S. aureus* biofilm, PHMB showed no statistically significant reduction within 24 h compared to an untreated control (Figures 2B,C; *p* = 0.90/0.93), while OCT/PE managed a statistically significant low reduction of 0.83 ± 0.23 (Figure 2B; *p* = 0.014) and 1.28 ± 0.32 log_10_ steps (Figure 2C; *p* = 0.0002).

A higher efficacy could be observed against *P. aeruginosa* biofilms. Both antiseptics induced a statistically significant reduction within 24 h of treatment compared to the control, whereby OCT/PE achieved 2.68 ± 0.46 log_10_ steps (*p* = 0.0008) and PHMB 2.97 ± 0.59 log_10_ steps (Figure 2A; *p* = 0.0002).

### Anti-biofilm Efficacy (hpBIOM) Under Prolonged Exposure (up to 72 H) and/or Increased Substance Volume (1 ml)

Prolonged exposure times of up to 72 h for 0.3 ml OCT/PE and PHMB increased bacterial reduction of all three pathogens with a continuous decrease in bacterial counts yielding highest reductions after 72 h of exposure compared to the untreated control (Figures 2A–C). For MRSA and MSSA, OCT/PE reached higher overall reduction rates than PHMB after 72 h (MRSA: 4.45 ± 1.73 vs. 0.96 ± 0.79 log_10_; MSSA – 2.19 ± 0.29 vs. 0.97 ± 0.79 log_10_; Figures 2B,C). Against *P. aeruginosa* biofilms, PHMB achieved higher reduction rates than OCT/PE after 72 h (4.23 ± 1.95 vs. 3.54 ± 1.34 log_10_; Figure 2A). In case of antimicrobial hypochlorous irrigation solutions, a prolonged exposure with 0.3 ml showed no improved effect (Figure 1).

**Figure 3:**
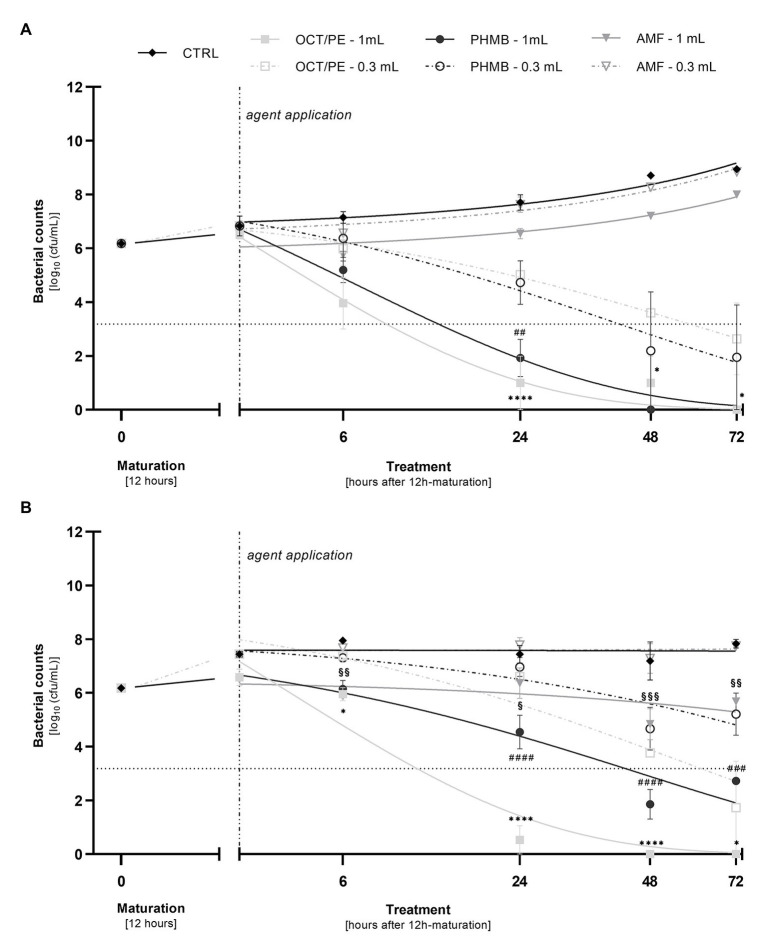
Comparison of the reduction of *P. aeruginosa* **(A)** and MRSA **(B)** in the biofilm model (hpBIOM) between 0.3 and 1.0 ml of tested antimicrobial substances in a prolonged exposure of up to 72 h. Graphs depict bacterial counts (in log_10_ cfu/ml) after treatment with octenidine-dihydrochloride/phenoxyethanol (OCT/PE), polyhexanide (PHMB), or 0.2% sodium hypochlorite (AMF) compared to an untreated control (CTRL). Values are expressed as means ± *sem* and a significant increase in reduction under 1.0 ml substance volume is expressed as ^*^p ≤ 0.05, ^**^p ≤ 0.01, ^***^p ≤ 0.001, or ^****^p ≤ 0.0001 (* for PHMB; # for OCT/PE, and § for AMF). Dotted horizontal line indicates the cut-off for a high antimicrobial efficacy (≥3 log_10_ reduction steps). Dashed vertical line indicates onset of treatment with the different antimicrobial agents after 12 h of biofilm maturation. All experiments were performed in triplicates.

When increasing the substance volume per treatment to 1.0 ml, both OCT/PE and PHMB demonstrated a significant increase of bacterial reduction within 24 h compared to 0.3 ml (Figure 3). OCT/PE achieved a nearly complete eradication of MRSA and *P. aeruginosa* after 24 h and especially against MRSA biofilms, it showed a significantly higher reduction than PHMB (5.64 ± 0.53 vs. 1.63 ± 0.63 log_10_; *p* < 0.0001; Figure 3B). For hypochlorous solutions, an increase in volume did not result in an increase in antimicrobial efficacy within 24 h (Supplementary Table 2).

The highest anti-biofilm efficacy was observed under the combination of prolonged exposure and increased substance volume of 1 ml (Figures 3A,B). OCT/PE managed to completely eradicate both MRSA and *P. aeruginosa* biofilms after 72 h. In case of *P. aeruginosa* biofilms, PHMB achieved full eradication before OCT/PE, after only 48 h (Figure 3A), while against MRSA it proved significantly less effective (2.73 log_10_ reduction steps less than OCT/PE after 72 h; *p* = 0.0004), not achieving complete eradication and even demonstrating a certain regrowth between 48 and 72 h (Figure 3B).

In terms of the antimicrobial hypochlorous solutions, the highest concentrated product (AMF; 0.2% sodium hypochlorite) demonstrated a low bacterial reduction against MRSA biofilm of 1.35 ± 0.58 log_10_ steps (*p* = 0.0016) after 48 h. However, regrowth between 48 and 72 h could be observed (Figure 3B) as well.

### Scanning Electron Microscopy of the hpBIOM

**Figure 4:**
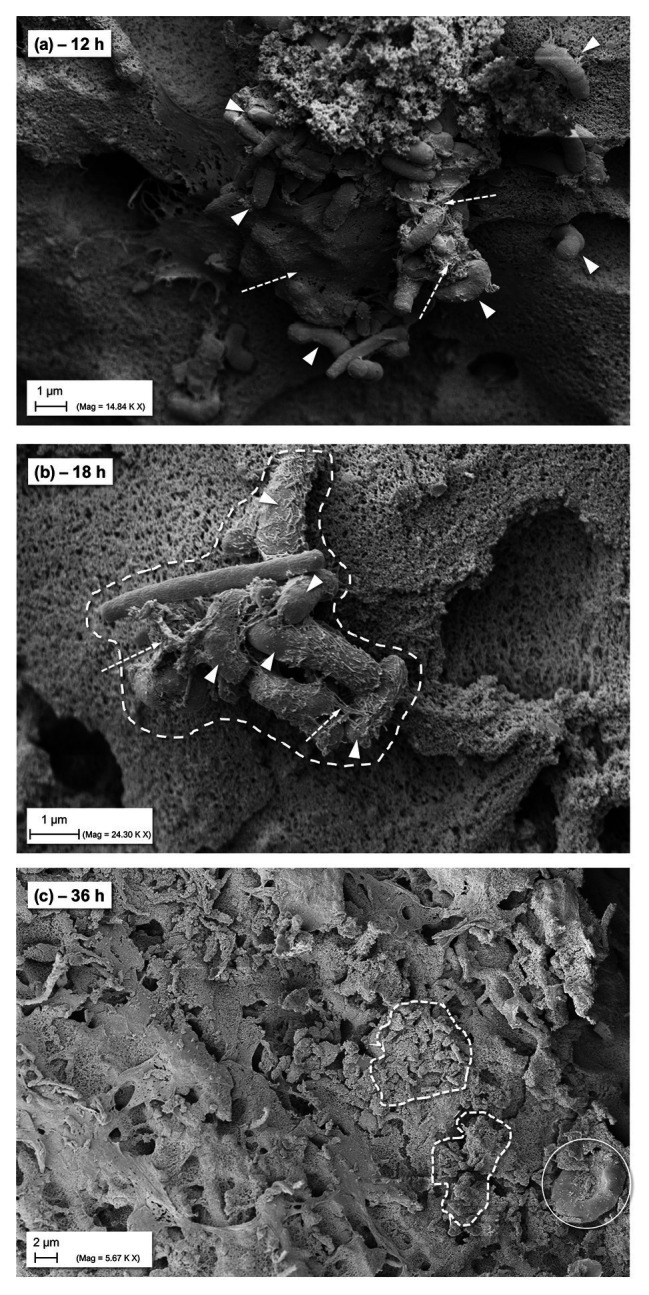
SEM images of general biofilm maturation and development in the hpBIOM (here *P. aeruginosa*) without treatment (CTRL). **(A)** Formed microcolony in a 12 h maturated biofilm with bacterial attachment and initially formed extracellular polymeric substance (EPS)/glycocalyx (arrows with dashed lines); arrowheads indicate bacteria. **(B)** Eighteen hours matured biofilm: dashed framing circles bacterial microcolony with single bacteria (arrowheads) connected by EPS/glycocalyx (arrows with dashed lines). **(C)** Surface view of 36 h matured biofilm with densely integrated and glycocalyx-surrounded bacteria (dashed framing) and human cells (white circle; erythrocyte).

The monitoring and visualization of the hpBIOM using SEM (representative imaging provided from *P. aeruginosa* biofilms; Table 2) showed a good maturation and development of the biofilm with initial microcolony formation and subsequent development of EPS/glycocalyx, encapsulating bacteria (Figure 4) within 18 h. After 36 h of maturation, a densely formed surface of interconnected, polymerized fibrins with encapsulated bacteria and densely distributed EPS can be observed.

**Figure 5:**
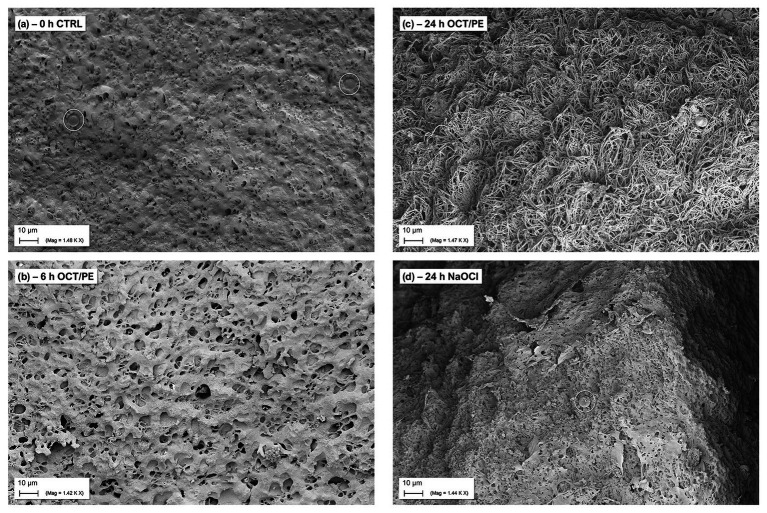
SEM visualization of biofilm surface alteration in the hpBIOM (here *P. aeruginosa*) under treatment with antiseptics and antimicrobial wound irrigation solutions. **(A)** Densely connected surface structure of a 12 h-maturated biofilm before treatment, white circles depict human erythrocytes. **(B)** After 6 h of treatment with OCT/PE: surface structure appears less compact with several holes as potential entry points. **(C)** After 24 h of treatment with OCT/PE: surface is deranged and “broken-open” into a loosened structure with fine filaments, readily penetrable. **(D)** After 24 h of treatment with <0.08% NaClO: remaining densely connected surface structure, no visible penetration, additional build-up superficial EPS structures (white circle depicts human erythrocyte).

Under treatment with hypochlorous solutions and antiseptics compared to no treatment, a loss in surface density of the biofilm model can be observed with increasing treatment time of the antiseptic OCT/PE (Figure 5). After 6 h of treatment, the biofilm surface shows an increased number of holes and less integrity compared to the initial untreated surface structure (Figure 5B). After 24 h of treatment, the surface is deranged and “broken-open” into a loosened structure with fine filaments, readily penetrable by OCT/PE (Figure 5C). After 24 h of treatment with <0.08% NaClO, the surface structure remained densely connected without visual loosening of surface structure or holes (Figure 5D). Additionally, newly build-up superficial EPS structures appeared, covering some area of the biofilm surface structure.

## Discussion

Biofilms pose a great challenge in chronic wound care and are a major factor for wound chronicity and impaired healing (James et al., 2008; Percival and Suleman, 2015; IWII, 2016; Percival, 2017; Schultz et al., 2017). Adequately addressing this challenge by improving knowledge and developing precise, comprehensive and new therapeutic strategies, including accurate agent efficacy profiles, is an important research goal in modern wound management (Schultz et al., 2017; Schwarzer et al., 2019; Stoffel et al., 2020).

Due to the extensive resilience of microorganisms residing within polymicrobial communities protected by EPS, rigorous and highly efficient anti-biofilm regimens are necessary. A form of repetitive, effective debridement represents the fundamental base of current anti-biofilm strategies (Wolcott et al., 2010; IWII, 2016; Schultz et al., 2017). Debridement alone, however, is insufficient for complete biofilm eradication (Schwartz et al., 2014; Schultz et al., 2017), as is the lone application of (even highly efficient) antimicrobial and antiseptic agents, making a combination of both approaches the current gold standard (Hoiby et al., 2015; Koo et al., 2017; Schultz et al., 2017). However, reported data on efficacy of available antimicrobial and antiseptic agents varies greatly depending on various factors and to date no agent can be clearly favored as being best suited for the treatment of chronic wound biofilms (Schultz et al., 2017; Schwarzer et al., 2019). Foremost, heterogeneity in study design/experimental setup and lacking transferability from adequate *in-vitro* to *in-vivo* studies and the clinical reality have been identified as a main limitation in current research (Schwarzer et al., 2019; Stoffel et al., 2020).

Another major concern is the sometimes liberal extrapolation of results in basic “non-challenging” test scenarios (e.g., antimicrobial efficacy in planktonic assays or biofilms grown on plastic surfaces without introduction of organic load) to clinical “real-world” situations. The significant differences in efficacy between a standard planktonic (DIN EN 13727; DIN, 2013) and *in-vitro* biofilm assay as well as between different forms of biofilm assays have been demonstrated before (Brackman and Coenye, 2016; Johani et al., 2018; Shukla et al., 2020) and could be reproduced in this study (Figures 2A–C) when comparing reduction rates of QSM to hpBIOM. Where bacteria in a planktonic state are easily eradicated by the antiseptic OCT/PE and the PHMB-based antimicrobial irrigation solution within 30 min, reduction rates are significantly reduced in the more complex hpBIOM (Figures 2A–C) which introduces organic challenge faced by antimicrobial agents in human biofilm-burdened chronic wounds (higher protein load, hard-to-penetrate EPS structure, immune cells, dormant bacteria, etc.). These results emphasize the dependency of an agent’s performance based on the environment it acts in and are calling for more comprehensive testing of agents before recommendations can be declared.

Most static and liquid-flow-based *in-vitro* models are limited to factors such as growth on plastic surfaces and lack adequate organic conditions reflecting a wound environment, let alone the heterogenous, individual conditions in human chronic wound biofilms (Brackman and Coenye, 2016). The development of the hpBIOM (Figures 4A–C; Besser et al., 2019, 2020) aimed to narrow the gap between *in-vitro* and *in-vivo* biofilm research and provides a translational approach. The use of a complex biofilm model based on human material, including plasma and immune cells, addresses the interactions of microbial biofilms with the human wound micro-environment (James et al., 2008; Percival et al., 2015), as well as the relevant loss in efficacy of antiseptics and antimicrobials under challenging conditions (Rembe et al., 2018; Radischat et al., 2020).

This is especially true for newly introduced commercial agents, to provide comprehensive data on efficacy profiles in complex test scenarios and better estimate performance in *in-vivo* and in clinical situations. The presented work aimed to provide such data for commercially available chlorine-based agents (HOCl, NaOCl) and included the current “best-practice” antiseptic/antimicrobial agents as base of reference (OCT/PE and PHMB-based irrigation solution).

The presented results on antimicrobial hypochlorous wound irrigation solutions thereby highlight the necessity for complex evaluation models, as well as the careful distinction between agent classes. Generally, differentiation between an antiseptic agent and an antimicrobial wound irrigation solution is important: an antiseptic agent is a highly effective, chemical agent designated to eliminate microorganisms *via* a direct, pharmacological mode of action. An antimicrobial irrigation solution, on the other hand, has a primarily mechanistic approach, reducing microorganisms by means of dilution, mechanical detachment *via* washing off microorganisms from surfaces such as wounds and additional effects, such as lowering surface tension, pH modulation, preservation, and antimicrobial efficacy depending on nature and concentration of added active ingredient. Differentiation between these kinds of solutions is key for therapeutic implications, indications, and correct application in clinical practice. Otherwise, misguided expectations of, for example, a sufficient antibacterial or anti-biofilm effect, lead to insufficient local treatment. While antiseptics mostly undergo a more rigorous evaluation process regarding antimicrobial efficacy before commercial introduction, irrigation solutions are often licensed as medical devices with certain additives but a primary mechanistical mode of action. That makes licensing legitimately easier, however, classification in the field of anti-infective (antimicrobial, antiseptic, and anti-biofilm) agents reasonably more complicated since comprehensive efficacy evaluations are not provided. Oftentimes, antimicrobial modes of action are transferred from general concepts and similar agents or products without actually providing data, or only very basic, on the product itself in its specific formulation (considering concentration, pH, volume, etc.). In case of hypochlorous wound irrigation solutions, the generalized transferred concept is the “oxidative burst,” a defensive mechanism used by the human innate immune system, specifically neutrophils, *via* the conversion of H_2_O_2_ to HClO, a bactericidal agent, using the enzyme myeloperoxidase (Wang et al., 2007). While this effect might be highly efficient in endogenous pathogen defense, differences such as local concentrations, duration of exposure, endogenous production as needed, and other factors need to be accounted for if comparing endogenously produced HClO and externally administered HClO formulations. Such extrapolations often result in insufficient differentiation of antiseptics and antimicrobial irrigation solutions in wound care regarding their efficacy, with the potential risk of insufficient local anti-infective and anti-biofilm treatment. For these reasons, clearly and specific evaluated antimicrobial and anti-biofilm efficacy profiles for antiseptics as well as antimicrobial irrigation solutions in test setups “as-close-as-possible” to real life scenarios are necessary.

In a previous publication (Severing et al., 2019), we demonstrated and discussed the differences between commercially available hypochlorous wound irrigation solutions depending on agent concentration, pH-value, and test setup against planktonic bacteria, whereby LVX, KSL, and especially AMF showed a high short-term efficacy on planktonic bacteria. The results of the now presented work are based on these preliminary evaluations and extend them in terms of potential anti-biofilm efficacy. Unfortunately, no efficacy of either agent could be detected in the human biofilm model (hpBIOM) over the course of 72 h (Figure 1). The antiseptic reference agent OCT/PE and the PHMB-based irrigation solution, on the contrary, achieved significantly higher reductions of microorganisms (Figures 2A–C) and visual breaking open of biofilm structures (Figures 5A–C). In contrast to our earlier publication (Severing et al., 2019), hypochlorous agents not only failed to reduce microbial counts in a biofilm setup but also in the planktonic method (QSM; Figure 1). This is however most likely attributed to the experimental setup with a deliberately chosen lower “agent to microbial test suspension”-ratio in the planktonic QSM (1:5 compared to 8:1 in standards), to unify the ratio in both models (QSM and hpBIOM). OCT/PE and PHMB, however, still achieved complete eradication of all tested planktonic microorganisms within 6 h in the QSM (Figures 2A–C) even in low ratio setups. In a planktonic setup with higher volumes of hypochlorous irrigation solutions, higher reduction rates were achieved (Severing et al., 2019). These results standing in contrast to earlier studies on the efficacy of chlorine-based and comparable agents, at the same time underline the necessity for careful distinction of agent and solution formulations, as well as test settings. The discrepancies in studies evaluating different forms of chlorine-based solutions, reporting higher efficacies than reported here, mainly derive from the vast heterogeneity of study designs. Many *in-vitro* studies used stationary biofilms on plastic surfaces without organic challenge (D’Atanasio et al., 2015; Day et al., 2017; Romanowski et al., 2018), even though the relevance of such challenge has been widely acknowledged (Brackman and Coenye, 2016; Sato et al., 2019). Especially the influence of high and differential protein loads have been described as a major influential factor of antimicrobial efficacy (Kapalschinski et al., 2017; Radischat et al., 2020) and a study by Johani et al. (2018) directly demonstrates the loss of efficacy of relevant antimicrobial substances in short term application on *in-vitro* vs. *in-vivo* models, also for hypochlorous agents.

Also, as outlined earlier, the exact composition of solutions needs to be considered, differentiating between solutions containing mainly sodium-hypochlorite, hypochlorous acid, or both, as well as the concentration and pH of solution. Our previous publication demonstrates the increased efficacy of higher vs. lower concentrated chlorine-based solutions (Severing et al., 2019) on planktonic bacteria. The same pattern can be observed against biofilms in the here presented results, where the higher concentrated AMF (0.2% NaClO) is the only tested chlorine-based agent exhibiting any anti-biofilm effect (Figure 3B). However, this effect only occurs under increased volume and extended exposure. Compared to the antiseptic OCT/PE or the PHMB solution, the effect is still negligible and even though significantly increased compared to lower volumes, probably irrelevant in clinical practice. An additional influential aspect to be considered is the pH of evaluated solutions: more extreme acidic or alkaline preparations prove more effective (Percival et al., 2014; D’Atanasio et al., 2015; Wiegand et al., 2015; Day et al., 2017; Severing et al., 2019). Agents with acidic pH values in the work of D’Atanasio et al. (acidic solution with pH < 3) or Day et al. (acidic solution with pH of 5.5) or alkaline pH values (AMF with pH of 9.5), achieved effective microbial reductions compared to rather neutral pH ranges (pH of 6.5–8.7; e.g., LVX and KSL).

Further relevant aspects influencing the efficacy of especially antimicrobial wound irrigation solutions are substance volume and mechanical detachment under actual irrigation. To investigate whether an increase in substance volume would yield an improved anti-biofilm effect of hypochlorous solutions, the volume capabilities of the hpBIOM were exhausted to administer as much agent as possible (increasing the volume >3-fold to 1.0 ml). The reference substances OCT/PE and PHMB exhibited a significant increase in anti-biofilm efficacy, with OCT/PE and PHMB achieving complete eradication of *P. aeruginosa* (Figure 3A) and OCT/PE also eradicating MRSA biofilms within 72 h. The only hypochlorous solution, demonstrating an increase in efficacy compared to the lower volume was AMF (Figures 3A,B) with LVX and KSL showing no effect (Supplementary Table 2). Also, in case of AMF, the increased effect compared to the lower volume is rather irrelevant with at best 1.35 ± 0.58 log_10_ reduction compared to initial bacterial counts within 72 h (Figure 3B). For the reference substances on the contrary, the increase in administered volume as well as a prolonged exposure not only demonstrates a statistical significance regarding efficacy improvement but also proves clinically relevant by achieving the high reduction of bacterial counts (≥3 log_10_ reduction steps; Figures 3A,B) generally demanded from antiseptics under challenging conditions (DIN, 2013). This underlines the dose-dependency and differential approach antiseptics and antimicrobial irrigation solutions apply (“pharmacological” vs. “mechanical”) in terms of antimicrobial efficacy. The relevance of additional aspects such as dilution, mechanical detachment/debridement, and reduction in surface tension to the overall antimicrobial and cleansing effects of especially wound irrigation solutions reported in other studies (Kammerlander et al., 2011; Assadian et al., 2018) is indirectly highlighted here. On the other hand, this aspect needs to be accounted for as a limitation to this study and the here reported results: the intention of this work was to evaluate the anti-biofilm effect of the antimicrobial hypochlorous agents within antimicrobial wound irrigation solutions compared to a PHMB-based irrigation solution and the antiseptic OCT/PE. Additionally, the approach of simple increase in volume and duration of application to ameliorate antimicrobial efficacy of hypochlorous agents was to be evaluated, however, demonstrated no relevant improve in performance in contrast to PHMB-based solutions or OCT/PE (Figures 3A,B). Nevertheless, in clinical usage, the mechanical detachment effect and repetitive application, that cannot be adequately reproduced in the experimental approach used here, may result in an increased reduction of microbial and even biofilm burden. Naturally, concentration as well as applied volume cannot be infinitely increased due to potential limiting side-effects and toxicity, which needs to be acknowledged in the evaluation of antimicrobial and antiseptic agents. Thereby, especially highly potent agents exert a higher cytotoxicity potential, whereby antimicrobial efficacy and cytotoxicity rise directly proportional to each other as demonstrated in several earlier studies (Hirsch et al., 2010; Severing et al., 2019). Herein lies another potential benefit of irrigation solutions with antimicrobial additives such as hypochlorous agents: while the sole antimicrobial efficacy is relevantly less compared to other agents, the likewise relevantly lower cytotoxicity allows for a far higher safely administrable substance volume, potentially making up for the lower direct antimicrobial effect *via* dilution an mechanical cleansing. This is most certainly the case for the decontamination and decolonization of chronic wounds, where the regenerating wound bed is to be protected from negative toxic influence and only a somewhat lower antimicrobial effect is desired.

The reported results highlight the necessity to apply antimicrobial irrigation solutions and antiseptics for a sufficient amount of time, to achieve biofilm penetration and effectively reduce bacterial counts (if used alone). The difficulty of biofilm penetration observed here indirectly confirms the current “state of the art” treatment approach of combining local antimicrobial treatment with debridement to mechanically break open dense biofilm structures and facilitate antimicrobial penetration. Generally, data on agent specific efficacy profiles need to be extended and investigated in more comprehensive and complex models to avoid confusion and misconception. Agent classification and situation-based therapeutic regimens are needed with distinguishing between highly potent antiseptics, more and less effective antimicrobial irrigation solutions and neutral irrigation solution in modern complex wound management.

Low-dosed, (near-)neutral pH hypochlorous wound irrigation solutions seem unsuitable for sole and first-line anti-biofilm treatment based on the inability to reduce bacterial counts in this complex hpBIOM. Naturally, these results should be translated to clinical practice with caution since above discussed limitations as well as further aspects such as the use of mono-species biofilms, short maturation times (12 h), and the unclear effect of repetitive extensive volume irrigation, still apply. However, more mature and multi-species biofilms would exhibit even higher bacterial resilience, as partly already demonstrated in another work of our research group (Besser et al., 2020) and, therefore, pose an even greater challenge for hypochlorous irrigation solutions. The anti-biofilm capacities in terms of eradication and antimicrobial effect of such wound irrigation solutions are, therefore, limited. The potential dilution and mechanical detachment of early, loosely attached microorganisms combined with the additive effect of a low-ranging antimicrobial efficacy, rather support hypochlorous wound irrigation solutions as suited for decontamination and decolonisation of acute and chronic wounds and, therefore, prevention of (re-)contamination/infection, rather than primary treatment of mature biofilms.

## Data Availability Statement

The datasets used and/or analyzed during the current study are available from the corresponding author on reasonable request or are available as supplementary data files.

## Author Contributions

JDR, LH, and ES designed the study. JDR and LH performed experiments, data analysis, and drafted the figures. MB and IP helped with data analysis, interpretation, and experimental setup. IP and LH performed SEM imaging. JDR, LH, and ES drafted and finalized the manuscript. All authors contributed to the article and approved the submitted version.

### Conflict of Interest

The authors declare that the research was conducted in the absence of any commercial or financial relationships that could be construed as a potential conflict of interest.

## Figures and Tables

**Table:** 

Term	Definition
AMF	Actimaris® forte
CaCl_2_	Calcium chloride
CFU	Colony forming units
CSA	Casein/soy peptone agar
CTRL	Control
DIN	German institute for standardization
DRK	German red cross
DSMZ	German Collection of Microorganisms and Cell Cultures
ddH_2_O	Double distilled water
ECM	Extracellular matrix
EPS	Extracellular polymeric substance
HCL	Hydrochloride
HClO	Hypochlorous acid
hpBIOM	Human plasma biofilm model
KSL	Kerrasol®
LVX	Lavanox®
MRSA	Methicillin-resistent *S. aureus*
MSSA	Methicillin-susceptible *S. aureus*
NaClO	Sodium hypochlorite
NaNO_2_	Sodium nitrite
OCT/PE	Octenidine dihydrochloride/phenoxyethanol
OsO_4_	Osmium tetroxide
PBS	Phosphate buffered saline
PHMB	Polyhexamethylene-biguanide
PVP	Polyvinylpyrrolidone
QSM	Quantitative suspension method
RT	Room temperature
SEM	Scanning electron microscopy
*sem*	Standard error of the mean
SDS	Sodium dodecyl sulfate
