# Supplementary material for: Antimicrobial Hypochlorous Wound Irrigation Solutions Demonstrate Lower Anti-biofilm Efficacy Against Bacterial Biofilm in a Complex in-vitro Human Plasma Biofilm Model (hpBIOM) Than Common Wound Antimicrobials
Source: Front Microbiol. 2020 Oct 9;11:564513. doi: 10.3389/fmicb.2020.564513 (PMC7583357; doi:10.3389/fmicb.2020.564513)
Supplement: Supplementary file 1 [file Data_Sheet_1.docx]

**Supplementary Data**

**Supplementary Table S1** – Reduction rates in the qualitative suspension method (QSM) against planktonic bacteria (*S. aureus*, MRSA and *P. aeruginosa*) of tested substances (0.3 mL) after 0.5, 2, 6 and 24 hours (48 hours and 72 hours were not tested, due to either complete reduction or clear ineffectiveness). Negative values represent an increase in bacterial counts. Values are expressed as mean ± *sem* (in log_10_ cfu/mL).

| **Table S1 - Quantitative Suspension Method** | | | | | |  |  |
| --- | --- | --- | --- | --- | --- | --- | --- |
|  |  | *S. aureus* planctonic | |  |  |  |  |
|  |  | **0.3 mL** |  |  |  |  |  |
|  |  | **0.5 h** | **2 h** | **6 h** | **24 h** | **48 h** | **72 h** |
| **CTRL** |  | -0.57±0.09 | -0.48±0.10 | -0.80±0.15 | -3.43±0.13 | - | - |
| **OCT/PE** |  | 6.18±0.00 | 6.18±0.00 | 6.18±0.00 | 6.18±0.00 | - | - |
| **PHMB** |  | 4.82±1.36 | 4.81±1.37 | 6.18±0.00 | 6.18±0.00 | - | - |
| **LVX** |  | -0.37±0.04 | -0.32±0.06 | -0.62±0.11 | -3.30±0.17 | - | - |
| **KSL** |  | -0.25±0.05 | -0.49±0.10 | -0.66±0.13 | -3.12±0.21 | - | - |
| **AMF** |  | -0.33±0.03 | -0.32±0.03 | -0.50±0.07 | -3.00±0.31 | - | - |
|  |  |  |  |  |  |  |  |
|  |  | *MRSA* planctonic | |  |  |  |  |
|  |  | **0.3 mL** |  |  |  |  |  |
|  |  | **0.5 h** | **2 h** | **6 h** | **24 h** | **48 h** | **72 h** |
| **CTRL** |  | -0.86±0.00 | -0.71±0.15 | -0.87±0.10 | -2.60±0.43 | - | - |
| **OCT/PE** |  | 6.18±0.00 | 6.18±0.00 | 6.18±0.00 | 6.18±0.00 | - | - |
| **PHMB** |  | 6.18±0.00 | 6.18±0.00 | 6.18±0.00 | 6.18±0.00 | - | - |
| **LVX** |  | -0.29±0.26 | -0.32±0.19 | -0.47±0.25 | -2.49±0.45 | - | - |
| **KSL** |  | -0.37±0.22 | -0.34±0.24 | -0.50±0.25 | -2.48±0.43 | - | - |
| **AMF** |  | -0.32±0.27 | -0.31±0.20 | -0.37±0.24 | -2.36±0.44 | - | - |
|  |  |  |  |  |  |  |  |
|  |  | *P. aeruginosa* planctonic | |  |  |  |  |
|  |  | **0.3 mL** |  |  |  |  |  |
|  |  | **0.5 h** | **2 h** | **6 h** | **24 h** | **48 h** | **72 h** |
| **CTRL** |  | -0.74±0.03 | -0.22±0.31 | -0.55±0.18 | -4.01±0.07 | - | - |
| **OCT/PE** |  | 4.77±1.41 | 6.18±0.00 | 6.18±0.00 | 6.18±0.00 | - | - |
| **PHMB** |  | 5.25±0.93 | 6.18±0.00 | 6.18±0.00 | 6.18±0.00 | - | - |
| **LVX** |  | -0.64±0.01 | -0.28±0.03 | 0.04±0.04 | -3.87±0.04 | - | - |
| **KSL** |  | -0.54±0.07 | -0.31±0.03 | 0.19±0.34 | -3.89±0.06 | - | - |
| **AMF** |  | -0.57±0.08 | -0.34±0.03 | -0.09±0.07 | -3.85±0.06 | - | - |

**Supplementary Table S2A & S2B** – Reduction rates in the human plasma biofilm model (hpBIOM) against *S. aureus*-, MRSA- or *P. aeruginosa*-biofilms of tested substances, either **(A)** 0.3 or **(B)** 1.0 mL volume) after 0.5, 2, 6, 24, 48 or 72 hours (0.5 hours and 2 hours were not tested for the increased volume of 1.0 mL). Negative values represent an increase in bacterial counts. Values are expressed as mean ± *sem* (in log_10_ cfu/mL).

| **Table S2A - hpBIOM (0.3 mL)** | | | |  |  |  |  |
| --- | --- | --- | --- | --- | --- | --- | --- |
|  |  | *S. aureus* biofilm | |  |  |  |  |
|  |  | **0.3 mL** |  |  |  |  |  |
|  |  | **0.5 h** | **2 h** | **6 h** | **24 h** | **48 h** | **72 h** |
| **CTRL** |  | -1.87±0.39 | -1.66±0.13 | -1.62±0.16 | -1.73±0.07 | -1.40±0.49 | -2.14±0.60 |
| **OCT/PE** |  | -1.52±0.16 | -1.48±0.16 | -1.06±0.15 | -0.45±0.25 | 2.28±0.61 | 2.19±0.29 |
| **PHMB** |  | -1.40±0.13 | -1.34±0.06 | -1.26±0.20 | -1.58±0.06 | -0.92±0.29 | 0.97±0.79 |
| **LVX** |  | -1.52±0.08 | -1.50±0.09 | -1.60±0.15 | -1.54±0.08 | -2.07±0.42 | -1.78±0.34 |
| **KSL** |  | -1.53±0.14 | -1.42±0.02 | -1.64±0.09 | -1.78±0.11 | -1.05±0.75 | -1.53±0.23 |
| **AMF** |  | -1.41±0.15 | -1.50±0.08 | -1.51±0.10 | -1.60±0.12 | -1.13±0.56 | -1.41±0.15 |
|  |  |  |  |  |  |  |  |
|  |  | *MRSA* biofilm | |  |  |  |  |
|  |  | **0.3 mL** |  |  |  |  |  |
|  |  | **0.5 h** | **2 h** | **6 h** | **24 h** | **48 h** | **72 h** |
| **CTRL** |  | -1.57±0.06 | -1.84±0.03 | -1.77±0.03 | -1.26±0.32 | -1.01±0.71 | -1.66±0.16 |
| **OCT/PE** |  | -1.59±0.05 | -1.61±0.10 | -1.21±0.25 | -0.43±0.14 | 2.41±0.12 | 4.45±1.73 |
| **PHMB** |  | -1.48±0.12 | -1.60±0.13 | -1.13±0.15 | -0.78±0.36 | 1.51±0.79 | 0.96±0.79 |
| **LVX** |  | -1.64±0.03 | -1.68±0.09 | -1.54±0.06 | -1.55±0.12 | -1.44±0.10 | -1.77±0.05 |
| **KSL** |  | -1.61±0.01 | -1.76±0.06 | -1.66±0.07 | -1.39±0.20 | -1.25±0.28 | -2.19±0.26 |
| **AMF** |  | -1.53±0.06 | -1.65±0.12 | -1.49±0.07 | -1.66±0.24 | -1.11±0.55 | -1.57±0.08 |
|  |  |  |  |  |  |  |  |
|  |  | *P. aeruginosa* biofilm | |  |  |  |  |
|  |  | **0.3 mL** |  |  |  |  |  |
|  |  | **0.5 h** | **2 h** | **6 h** | **24 h** | **48 h** | **72 h** |
| **CTRL** |  | -0.76±0.37 | -1.12±0.19 | -0.97±0.22 | -1.53±0.29 | -2.54±0.04 | -2.76±0.00 |
| **OCT/PE** |  | -0.57±0.49 | -0.29±0.43 | 0.19±0.62 | 1.16±0.49 | 2.57±0.79 | 3.54±1.34 |
| **PHMB** |  | -0.43±0.53 | -0.81±0.51 | -0.19±0.85 | 1.45±0.81 | 3.99±2.19 | 4.23±1.95 |
| **LVX** |  | -0.49±0.48 | -0.88±0.24 | -1.15±0.45 | -1.42±0.22 | -2.45±0.37 | -2.76±0.07 |
| **KSL** |  | -0.48±0.43 | -0.79±0.46 | -0.63±0.16 | -1.70±0.13 | -2.15±0.05 | -2.62±0.06 |
| **AMF** |  | -0.44±0.46 | -0.66±0.29 | -0.38±0.38 | -1.45±0.29 | -2.09±0.08 | -2.66±0.05 |

| **Table S2B - hpBIOM (1.0 mL)** | | | |  |  |  |  |
| --- | --- | --- | --- | --- | --- | --- | --- |
|  |  | *MRSA* biofilm | |  |  |  |  |
|  |  | **1.0 mL** |  |  |  |  |  |
|  |  | **0.5 h** | **2 h** | **6 h** | **24 h** | **48 h** | **72 h** |
| **CTRL** |  | - | - | -0.16±0.33 | -1.20±0.07 | -1.06±0.42 | -1.55±0.12 |
| **OCT/PE** |  | - | - | 0.22±0.24 | 5.64±0.53 | 6.18±0.00 | 6.18±0.00 |
| **PHMB** |  | - | - | 0.04±0.32 | 1.63±0.63 | 4.32±0.56 | 3.45±0.04 |
| **LVX** |  | - | - | -0.39±0.49 | -0.59±0.39 | -0.23±0.01 | 0.15±0.21 |
| **KSL** |  | - | - | -0.45±0.48 | -0.66±0.25 | -0.33±0.09 | -1.95±0.20 |
| **AMF** |  | - | - | 0.31±0.15 | -0.18±0.57 | 1.35±0.58 | 0.50±0.04 |
|  |  |  |  |  |  |  |  |
|  |  | *P. aeruginosa* biofilm | |  |  |  |  |
|  |  | **1.0 mL** |  |  |  |  |  |
|  |  | **0.5 h** | **2 h** | **6 h** | **24 h** | **48 h** | **72 h** |
| **CTRL** |  | - | - | -0.13±0.33 | -1.22±0.07 | -2.65±0.18 | -2.62±0.03 |
| **OCT/PE** |  | - | - | 2.22±0.96 | 5.18±1.00 | 5.18±1.00 | 6.18±0.00 |
| **PHMB** |  | - | - | 0.98±0.47 | 4.26±0.70 | 6.18±0.00 | 6.18±0.00 |
| **LVX** |  | - | - | 0.03±0.23 | -0.90±0.34 | -1.38±0.16 | -2.10±0.15 |
| **KSL** |  | - | - | 0.11±0.37 | -0.46±0.18 | -1.69±0.04 | -2.33±0.13 |
| **AMF** |  | - | - | 0.42±0.55 | -0.37±0.19 | -1.03±0.02 | -1.82±0.10 |
